# Five-meter walk test before transcatheter aortic valve replacement and 1-year noncardiac mortality

**DOI:** 10.1016/j.xjon.2022.08.003

**Published:** 2022-08-18

**Authors:** Toshinobu Kazui, Chiu-Hsieh Hsu, Mohammad Hamidi, Deepak Acharya, Madhan Shanmugasundaram, Kwan Lee, Arka Chatterjee, David Bull

**Affiliations:** a Division of Cardiothoracic Surgery, Department of Surgery, Banner University Medical Center-Tucson/University of Arizona, Tucson, Ariz; b Department of Surgery, Banner University Medical Center-Tucson/University of Arizona, Tucson, Ariz; c Department of Epidemiology and Biostatistics, Mel and Enid Zuckerman College of Public Health, The University of Arizona, Tucson, Ariz; d Division of Cardiology, Department of Medicine, Sarver Heart Center, Banner University Medical Center-Tucson/University of Arizona, Tucson, Ariz

**Keywords:** transcatheter aortic valve replacement, 5-m walk test, noncardiac mortality, 1-year mortality, AS, aortic valve stenosis, KCCQ12, Kansas City Cardiomyopathy Questionnaire, NYHA, New York Heart Association, STS-PROM, Society of Thoracic Surgeons predicted risk of mortality score, TAVR, transcatheter aortic valve replacement, TVT Registry, Transcatheter Valve Therapy Registry

## Abstract

**Objective:**

The purpose of this study is to assess whether the 5-m walk test is associated with 1-year mortality after transcatheter aortic valve replacement.

**Methods:**

Included in the analysis were 304 patients who received the 5-m walk test and underwent transcatheter aortic valve replacement from September 2012 to March 2019. They were classified into 3 groups based on their test score: ≤7, >7, and unable to walk. Preprocedure characteristics, postprocedure outcomes, and follow-up outcomes were compared between the groups.

**Results:**

For the 5-m walk test, 145 had a score ≤7 (Group N), 111 had a score >7 (Group S), and 48 were unable to walk (Group I). Average age in years was 80.2 ± 8.7 years in Group N, 81.2 ± 9.4 years in Group S, and 79.4 ± 9.2 in Group I (*P* = .23). The aortic valve mean gradient at discharge was 9.5 ± 4.1 mm Hg in Group N, 10.4 ± 5.5 mm Hg in Group S, and 8.2 ± 4.2 mm Hg in Group I (*P* = .05). The discharge survival was 97.2% in Group N, 96.4% in Group S, and 95.8% in Group I (*P* = .76). One-year survival was 92.8% in Group N, 84.1% in Group S, and 75% in Group I (*P* < .01) after adjusting for preprocedure characteristics. Noncardiac death was 5.1% in Group N, 13.1% in Group S, and 22.7% in Group I (*P* = .03). This indicates that the 5-m walk test was a risk factor for 1-year mortality. More specifically, a poor 5-m walk test score was associated with 1-year noncardiac mortality.

**Conclusions:**

The 5-m walk test score before transcatheter aortic valve replacement was associated with 1-year mortality, especially noncardiac mortality. It may help identify patients at high risk for 1-year mortality.

## Graphical abstract

**Figure:**
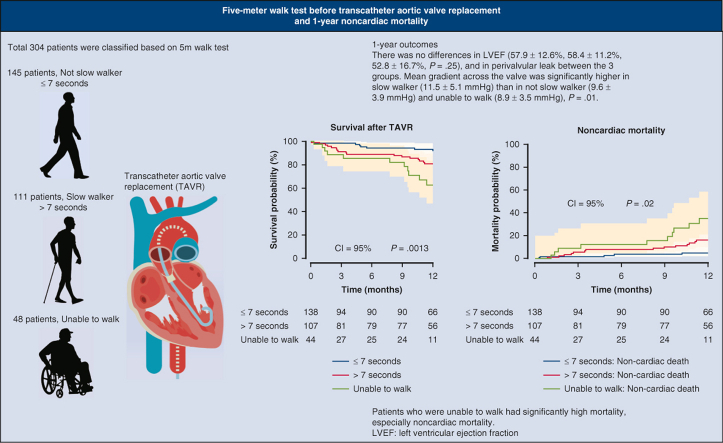

**Figure:**
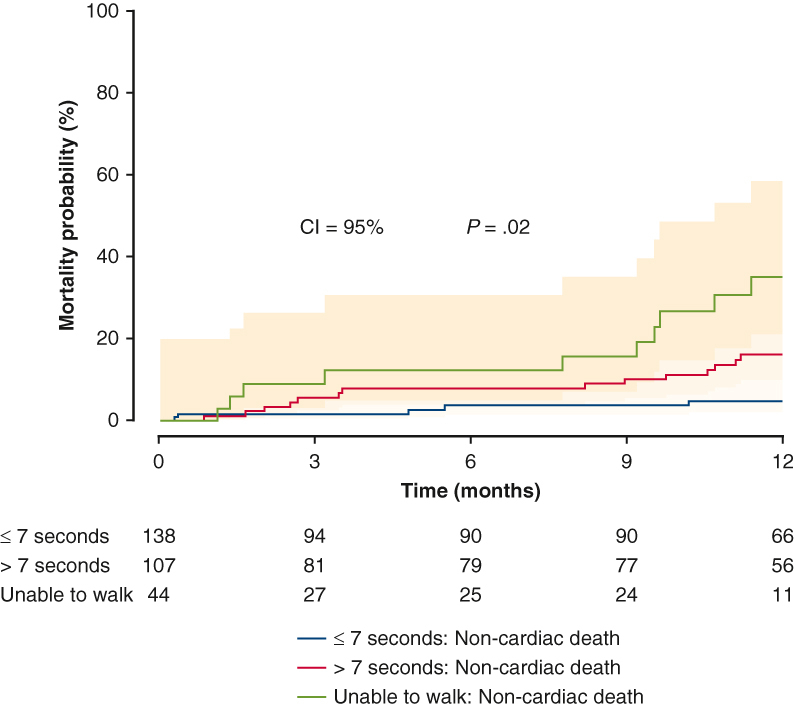
Pre-TAVR 5-m walk test score was associated with noncardiac mortality at 1 year.

**Central Message**

Pretranscatheter aortic valve replacement 5-m walk test was an independent risk factor for 1-year mortality despite improved aortic valve function.

**Perspective**

Despite good procedural outcomes at follow up, 1-year survival after TAVR for symptomatic severe aortic valve stenosis depended on a pre-TAVR 5-m walk test performance. Patients who were not able to ambulate or took more than 7 seconds had poorer survival than patients who took less than 7 seconds. This finding can help patient selection and management.

The transcatheter aortic valve replacement (TAVR) has become the standard therapy for all symptomatic severe aortic valve stenosis (AS).^1^^,^^2^ This makes quality measurement even more important. Currently, hospital performance metrics, such as 30-day mortality, are used as quality metrics.^3^^,^^4^ However, previous studies indicate that 30-day outcomes can lead to misclassification of the quality of a hospital's care. Longer than 30-day survival after TAVR may be a more accurate quality metric.^5^^,^^6^ The Centers for Medicare and Medicaid Services have also mandated that all TAVR procedures in the United States shall be captured in a prospective clinical registry with 1-year follow-up. Furthermore, the Centers for Medicare and Medicaid Services recommends continuation of the registry and the development of evidence linking procedure-related complications with longer-term patient health outcomes.

Although not a direct comparison of groups, a study of the Transcatheter Valve Therapy (TVT) Registry (created by the Society of Thoracic Surgeons and the American College of Cardiology) found that high-risk surgical patients had a worse post-TAVR 1-year survival rate than lower-risk surgical patients.^1^^,^^7^^,^^8^ Preprocedure frailty has been reported as among the risk factors of poor 1-year survival in high-risk surgical patients.^9^, ^10^, ^11^ Proposed surrogates for frailty include dominant handgrip strength, gait speed, the Katz activity of daily living survey, serum albumin, or skeletal muscle area derived by a preprocedural computed tomography scan.^9^^,^^10^^,^^12^ Likewise, the 5-m walk test is an easy test to assess gait speed as a preprocedure frailty assessment. Although the previous study demonstrated that frailty was the risk factor for poor survival,^13^ it was not clear if this indicated cardiac or noncardiac mortality. In other words, did TAVR-related complications or suboptimal hemodynamic outcomes (residual pressure gradient or perivalvular leak) contribute to the poor outcomes or were patients in a poor health status independent of cardiac conditions?

Given that TAVR has become the standard therapy for all AS patients, and that the field is also moving more toward accurate quality-control metrics, it is important to maintain good outcomes as the use of this procedure expands. Current guidelines recommend palliative care if the patient is at a high or prohibitive risk and if the patient is not expected to have a life expectancy with an acceptable quality of life for more than 1 year.^14^ Currently, there is lack of evidence regarding risk factors for poor 1-year survival after TAVR that includes the low-risk surgical patient population. Such knowledge is necessary to better tailor case management and ensure good long-term outcomes. Given accumulating evidence of poor survival in frail patients after TAVR, our hypothesis is that a preprocedure-related poor performance in the 5-m walk test and a preprocedure-related high Society of Thoracic Surgeons predicted risk of mortality (STS-PROM) score were risk factors for 1-year mortality after TAVR. The purpose of this study was to assess whether the 5-m walk test is associated with 1-year survival after TAVR.

## Methods

### Study Cohort

This was a single-center retrospective study. Our institutional database was used to identify patients undergoing TAVR for symptomatic AS between September 2012 and March 2019 with completed follow-up visits. We utilized TVT Registry variables for this analysis. We divided those patients into 3 groups based on their 5-m walk test score (ie, not a slow walker ≤7 seconds [Group N], slow walker >7 seconds [Group S], and unable to walk/immobile [Group I]) and compared outcomes. We selected 7 seconds as a cutoff because it was the median among those who could walk. We further performed additional analysis to confirm that the slow 5-m walk test affected the noncardiac survival in 1 year. We used 3 different cutoffs: 6 seconds (first quarter), 8.18 seconds (mean), and 9 seconds (third quarter). The primary outcome was 1-year mortality. The University of Arizona Institutional Review Board approved this protocol as a minimal risk retrospective study that did not require individual patient consent in accordance with the institutional guidelines for waiving consent (170656324A002, date of IRB approval: May 14, 2019).

### Procedure-Related Outcomes

The primary outcome was 1-year mortality, defined by the TVT Registry. The cause of death was reviewed individually. Secondary outcomes included discharge survival, intensive care unit stay, the New York Heart Association (NYHA) functional classification at 1 year, and the Kansas City Cardiomyopathy Questionnaire (KCCQ12) at 1 year.

### Frailty Assessment Before TAVR

Frailty assessment was performed by the 5-m walk test, the serum albumin level, anemia (hemoglobin level), and the KCCQ12. A slow 5-m walk test was defined as completing the test in >7 seconds. Seven seconds was used because it was the median number. Unable to walk/immobile was defined as an inability to complete the test due to physical inability (deconditioning, highly symptomatic from heart failure). We excluded the patients who did not undergo the 5-m walk test. That was during an early period of the TAVRs when we did not have a system to perform it during hospital evaluation for TAVR. We performed a subanalysis comparing Group N and Group S to assess if the 5-m walk test score has an influence on 1-year mortality.

### Statistical Analysis

Preprocedure-related characteristics, postprocedure outcomes, and follow-up outcomes were compared between the groups by the Kruskal-Wallis test for continuous variables and the Fisher exact test for categorical variables. Logistic regression was performed to compare each of the dichotomous follow-up outcomes controlling for patient characteristics that were significant between the groups derived from the 5-m walk test result (ie, STS-PROM, KCCQ12 overall, prior aortic valve procedure, home oxygen, coronary artery disease presentation, prior 2-week heart failure, prior 2-week NYHA functional class, prior cardiogenic shock, preoperative hemoglobin level, and preoperative total albumin level). Log-normal regression was performed to compare relative (%) difference in left ventricular ejection fraction (LEVF) and KCCQ12, respectively. To compare 1-year mortality between the groups, the Kaplan-Meier estimation was performed to derive unadjusted 1-year mortality. Cox regression was performed to derive and compare adjusted 1-year mortality adjusting for patient characteristics that were significantly associated with mortality. Schoenfeld score residuals were used to evaluate the proportional hazard assumption. Finally, subdistribution hazards regression was performed to compare cause-specific mortality.

## Results

A total of 356 patients who underwent TAVR, including alternative access, were included in the study. Patients whose preprocedural 5-m walk test was not documented were excluded from the analysis. A total of 304 were included in the analysis. Of these, 48% (n = 145) were in Group N, 36% (n = 111) were in Group S, and 16% (n = 48) were in Group I. Table 1 demonstrates a summary of preprocedure patient characteristics by 5-m walk test seconds in the 3 different groups. The median age of Group N, Group S, and Group I was 82.1 years (interquartile range [IQR], 75.7-86.7 years), and 60.2% of patients were men. Seventy percent of patients were in NYHA functional class III or IV. Sixty percent of patients had previous cardiac surgical procedures with a median STS-PROM of 4.0% (IQR, 3.0%-7.0%). The percentage of patients at a low risk was 37.1%, 44.6% were in the intermediate risk, and 18.3% were in the high-risk group.

**Table 1:** Summary of preprocedure-related patient characteristics by the 5-m walk test

Variable	≤7 sec (n = 145)	>7 sec (n = 111)	Unable to walk (n = 48)	*P* value∗
Age	80.2 ± 8.7	81.2 ± 9.4	79.4 ± 9.2	.23
Male	93 (64.1)	58 (52.3)	32 (66.7)	.10
STS risk score (%)	4.2 ± 2.5	5.5 ± 3.4	6.8 ± 5.4	**<.001**
Missing	5	5	0	
KCCQ12, overall	52.3 ± 25.2	40.5 ± 24.8	34.3 ± 22.8	**<.001**
Missing	4	5	3	
Weight (kg)	76.8 ± 18.1	76.2 ± 17.8	84.3 ± 21.1	.17
Missing	3	3	4	
Prior pacemaker	17 (11.7)	9 (8.1)	6 (12.5)	.57
Prior PCI	65 (44.8)	40 (36.0)	22 (45.8)	.31
Prior CABG	41 (28.3)	22 (19.8)	10 (20.8)	.28
Prior aortic valve procedure	29 (20.0)	38 (34.2)	24 (50.0)	**<.001**
Prior AV: Replace	9 (6.2)	10 (9.0)	2 (4.2)	.53
Prior MV: Repair	1 (0.7)	3 (2.7)	0 (0.0)	.38
Prior MV: Replace	3 (2.1)	1 (0.9)	0 (0.0)	.66
Prior medical history				
Stroke	23 (15.9)	18 (16.2)	7 (14.6)	1.00
PAD	36 (24.8)	32 (28.8)	14 (29.2)	.68
Smoker	8 (5.5)	5 (4.5)	7 (14.6)	.062
HTN	136 (93.8)	103 (92.8)	47 (97.9)	.49
DM	51 (35.2)	38 (34.2)	26 (54.2)	.042
Current dialysis	1 (0.69)	4 (3.60)	3 (6.25)	.062
Chronic lung disease	42 (29.0)	41 (36.9)	23 (47.9)	.050
Home oxygen	20 (13.8)	17 (15.3)	17 (35.4)	**.004**
Hostile chest	4 (2.8)	1 (0.9)	0 (0.0)	.47
Immune suppressive agent	8 (5.5)	10 (9.0)	5 (10.4)	.39
CAD presentation				**.049**
No angina	46 (31.7)	50 (45.1)	12 (25.0)	
Non-STEMI	0 (0.0)	1 (0.9)	0 (0.0)	
Stable angina	6 (4.1)	7 (6.3)	1 (2.1)	
Symptoms unlikely to be ischemic	88 (60.7)	49 (44.1)	31 (64.6)	
Unstable angina	5 (3.5)	4 (3.6)	4 (8.3)	
Prior MI	43 (29.7)	31 (27.9)	16 (33.3)	.80
When				.12
<30 d	3 (7.0)	6 (19.4)	4 (25.0)	
≥30 d	40 (93.0)	25 (80.7)	12 (75.0)	
Missing	102	80	32	
Prior 2-wk HF	112 (77.2)	100 (90.1)	38 (79.2)	**.020**
Prior 2-wk NYHA				**<.001**
Class I	34 (23.5)	14 (12.6)	12 (25.0)	
Class II	19 (13.1)	13 (11.7)	0 (0.0)	
Class III	76 (52.4)	53 (47.8)	21 (43.8)	
Class IV	16 (11.0)	31 (27.9)	15 (31.3)	
Prior cardiogenic shock	0 (0.0)	0 (0.0)	2 (4.2)	**.024**
Prior cardiac arrest	0 (0.0)	1 (0.9)	0 (0.0)	.52
AFib/flutter	52 (35.9)	49 (44.1)	24 (50.0)	.16
AV morphology				.41
Bicuspid	1 (0.7)	3 (2.7)	1 (2.1)	
Tricuspid	139 (95.9)	102 (91.9)	47 (97.9)	
Uncertain	5 (3.5)	5 (4.5)	0 (0.0)	
Unicuspid	0 (0.0)	1 (0.9)	0 (0.0)	
Valve in valve	11 (7.6)	11 (9.9)	4 (8.3)	.79
Preoperative hemoglobin (g/dL)	12.3 ± 1.9	11.4 ± 1.9	10.6 ± 2.3	**< .001**
Preoperative creatinine (mg/dL)	0.7 ± 0.6	0.8 ± 01.0	0.9 ± 1.0	.70
Preoperative total albumin (g/dL)	3.1 ± 0.6	2.9 ± 0.6	2.8 ± 0.7	**<.001**
Missing	1	3	0	
LVEF (%)	55.0 ± 13.1	53.3 ± 14.3	50.3 ± 14.2	.087
Missing	0	1	0	
LVESD (cm)	2.9 ± 0.9	2.9 ± 1.0	3.2 ± 1.3	.42
Missing	14	16	8	
LVEDD (cm)	4.3 ± 1.0	4.3 ± 0.9	4.3 ± 1.1	.93
Missing	9	14	4	

Values are presented as mean ± SD or n (%). *STS*, Society of Thoracic Surgeons; *KCCQ12*, Kansas City Cardiomyopathy Questionnaire; *PCI*, percutaneous coronary intervention; *CABG*, coronary artery bypass grafting; *AV*, aortic valve; *MV*, mitral valve; *PAD*, peripheral artery disease; *HTN*, hypertension; *DM*, diabetes mellitus; *CAD*, coronary artery disease; *STEMI*, ST-elevation myocardial infarction; *MI*, myocardial infarction; *HF*, heart failure; *NYHA*, New York Heart Association; *AFib*, atrial fibrillation; *LVEF*, left ventricular ejection fraction; *LVESD*, left ventricular end systolic dimension; *LVEDD*, left ventricular end diastolic dimension.

∗ Derived from Kruskal-Wallis test for continuous variables and Fisher exact test for categorical variables.

Patients in Group N had a significantly lower STS-PROM score, a better KCCQ12, a higher preoperative hemoglobin level, and a higher albumin level than in Group S or Group I. In addition, patients in Group N were less likely to have a history of prior aortic valve intervention and had lower rates of diabetes mellitus compared with Group S and Group I. There were no differences between the groups in terms of age, gender, race, ethnicity, other comorbidities, presentation of their coronary artery disease, history of myocardial infarction, NYHA functional class, preoperative creatinine level, and LEVF.

Postprocedure patient characteristics are shown in Table 2. Most Group N patients underwent moderate sedation versus general anesthesia compared with the other 2 groups. Patients in Group N had higher postprocedure hemoglobin levels, lower postprocedure creatinine levels, and shorter intensive care unit length of stay after TAVR. There were no differences in postprocedure perivalvular leak, mean aortic valve gradient, or discharge to survival.

**Table 2:** Summary of postprocedure patient characteristics by the 5-m walk test

Variable	≤7 sec (n = 145)	>7 sec (n = 111)	Unable to walk (n = 48)	*P* value∗
CPB use	4 (2.8)	3 (2.7)	0 (0.0)	.76
Anesthesia type				**<.001**
Combination	0 (0.0)	0 (0.0)	1 (2.1)	
General anesthesia	50 (34.5)	75 (67.6)	23 (47.9)	
Moderate sedation	95 (65.5)	36 (32.4)	24 (50.0)	
Post AI				.60
Mild	24 (28.6)	26 (37.7)	9 (37.5)	
Moderate	5 (6.0)	6 (8.7)	2 (8.3)	
None	55 (65.5)	37 (53.6)	13 (54.2)	
Missing	61	42	24	
Postprocedural mean AV gradient (mm Hg)	4.8 ± 2.6	5.8 ± 3.4	4.9 ± 2.9	.16
Missing	24	29	6	
Postoperative haemoglobin (g/dL)	10.0 ± 2.3	9.1 ± 1.9	8.1 ± 1.9	**<.001**
Missing	0	2	0	
Postoperative creatinine (mg/dL)	0.7 ± 0.7	1.1 ± 1.2	1.0 ± 1.2	**.017**
Missing	0	2	0	
AV mean gradient at discharge (mm Hg)	9.5 ± 4.1	10.4 ± 5.5	8.2 ± 4.2	.051
Missing	7	4	1	
PRBCs unit transfused	3.0 ± 4.1	2.9 ± 3.1	2.9 ± 3.2	.64
Missing	124	81	34	
ICU stay (h)	29.5 ± 30.8	44.0 ± 32.4	37.8 ± 37.2	**.001**
Missing	3	7	6	
DC survival	141 (97.2)	107 (96.4)	46 (95.8)	.76
CE occurred	49 (33.8)	38 (34.2)	18 (37.5)	.90

Values are presented as n (%) or mean ± SD. *CPB*, Cardiopulmonary bypass; *AI*, aortic valve insufficiency; *AV*, aortic valve; *PRBC*, packed red blood cells; *ICU*, intensive care unit; *DC*, discharge; *CE*, any complication event.

∗ Derived from Kruskal-Wallis test for continuous variables and Fisher exact test for categorical variables.

**Figure 1:**
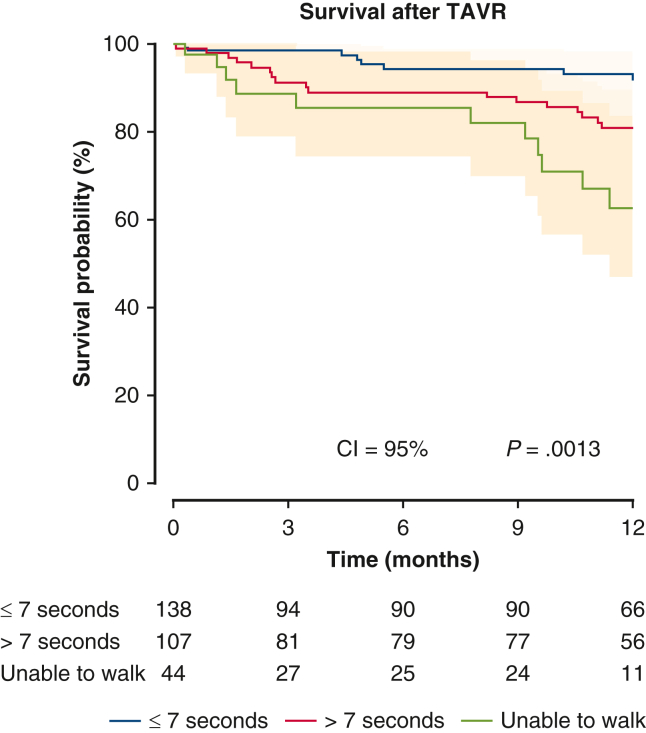
Survival after transcatheter aortic valve replacement (TAVR). The patients who walked 5 m in <7 seconds before TAVR had significantly better survival than patients who took >7 seconds and who were unable to walk before TAVR (*P* = .0013). Ninety five percent confidence limit was used.

**Figure 2:**
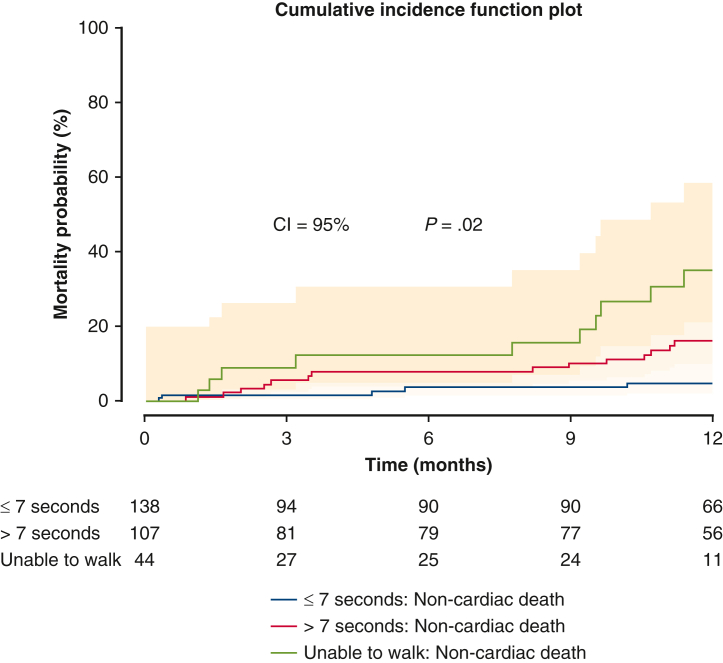
Noncardiac mortality of patients stratified by pretranscatheter aortic valve replacement 5-m walk test. The patients who were unable to walk before transcatheter aortic valve replacement had significantly worse noncardiac mortality than patients who walked 5 m in <7 seconds or who walked 5 m in >7 seconds (*P* = .03). Ninety five percent confidence limit was used.

Follow-up outcomes are summarized in Table 3. At 1-year follow up, Group N patients had the highest survival rate (92.8%) compared with 84.1% and 75.0% in Group S and Group I, respectively (*P* < .01) (Figure 1). No differences were noticed among the 3 groups in terms of NYHA functional class, aortic insufficiency, LVEF, and KCCQ12 overall score. The mean gradient across the valve was significantly higher in Group S than Group N or Group I. In addition, Group I had the worst noncardiac death rate at 1 year among Group N and Group S (Group N, Group S, Group I; 5.1% vs 13.1% vs 22.7%; adjusted hazard ratio, 4.06; 95% CI, 1.19-13.87; *P* = .03) (Figure 2 and Table E1). Table 4 demonstrates follow-up outcomes stratified by survival status. On survival status subanalysis, patients who died were more likely to be slow walkers, and they had significantly higher rates of a cardiac event (atrial fibrillation, conduction/native pacer, mitral valve reintervention, myocardial infarction, percutaneous coronary intervention, readmission-cardiac, readmission-heart failure, unplanned other cardiac surgery or intervention, or valve related readmission) and a noncardiac readmission event compared with the survival group. No difference was noticed between the 2 groups in terms of postaortic valve perivalvular leak. Regarding preoperative characteristics associated with follow-up survival, diabetes mellitus, atrial fibrillation/flutter, and unable to walk were risk factors for mortality at 1 year (Table 5).

**Table 3:** Follow-up outcomes by the 5-m walk test (1 year)

Variable	≤7 sec (n = 145)	>7 sec (n = 111)	Unable to walk (n = 48)	*P* value∗
Survival	128 (92.8) (n = 138)	90 (84.1) (n = 107)	33 (75.0) (n = 44)	**<.01**
Unadjusted odds ratio†	1.00	**0.45 (0.22-0.93)**	**0.25 (0.11-0.59)**	**<.01**
Adjusted odds ratio‡	1.00	0.72 (0.32-1.64)	**0.32 (0.12-0.85)**	.08
NYHA	n = 120	n = 76	n = 27	.10
I	95 (79.2)	55 (72.4)	16 (59.3)	
II	16 (13.3)	16 (21.1)	8 (29.6)	
III	8 (6.7)	4 (5.3)	1 (3.7)	
IV	1 (0.8)	1 (1.3)	2 (7.4)	
Unadjusted odds ratio (I)	1.00	0.69 (0.35-1.34)	0.38 (0.16-0.93)	.10
Adjusted odds ratio (I)	1.00	1.14 (0.53-2.49)	0.56 (0.20-1.56)	.40
AI	n = 117	n = 83	n = 28	.055
Mild	42 (35.9%)	23 (27.7%)	8 (28.6)	
Moderate	1 (0.9%)	6 (7.2%)	3 (10.7)	
None	51 (43.6%)	31 (37.4%)	13 (46.4)	
Trace	23 (19.7%)	23 (27.7%)	4 (14.3)	
Unadjusted odds ratio, mild or moderate	1.00	0.68 (0.37-1.26)	0.71 (0.29-1.76)	.43
Adjusted odds ratio, mild or moderate	1.00	0.60 (0.29-1.24)	0.71 (0.26-1.94)	.37
LVEF (%)	57.9 ± 12.6 (n = 119)	58.4 ± 11.2 (n = 84)	52.8 ± 16.7 (n = 28)	.25
Unadjusted relative risk§	1.00	1.01 (0.95-1.07)	0.91 (0.83-1.01)	.11
Adjusted relative risk	1.00	1.04 (0.97-1.11)	0.93 (0.84-1.03)	.08
KCCQ12, overall	74.5 ± 22.1 (n = 114)	65.5 ± 22.2 (n = 70)	48.0 ± 28.0 (n = 23)	**<.001**
Unadjusted relative risk	1.00	**0.88 (0.80-0.97)**	**0.65 (0.53-0.79)**	**<.0001**
Adjusted relative risk	1.00	**0.86 (0.77-0.95)**	**0.63 (0.51-0.78)**	**<.0001**
AV mean gradient (mm Hg)	9.6 ± 3.9 (n = 113)	11.5 ± 5.1 (n = 83)	8.9 ± 3.5 (n = 27)	**.01**
Unadjusted relative risk	1.00	**1.20 (1.07-1.35)**	0.93 (0.76-1.14)	**<.01**
Adjusted relative risk	1.00	**1.22 (1.08-1.38)**	1.00 (0.82-1.23)	**<.01**

Values are presented as n (%) or mean ± SD. *NYHA*, New York Heart Association; *AI*, aortic valve insufficiency; *LVEF*, left ventricular ejection fraction; *KCCQ12*, Kansas City Cardiomyopathy Questionnaire; *AV*, aortic valve.

∗ Derived from Kruskal-Wallis test for continuous variables and Fisher exact test for categorical variables for the comparison among the 3 groups; derived from logistic regression/log-normal regression for both unadjusted and adjusted *P* values assessing the effect of the 5-m walk test on each outcome.

† Odds ratio derived from logistic regression.

‡ Adjusted for patient characteristics significantly different between the 5-m walk test group in Table 1 (ie, Society of Thoracic Surgeons risk score, KCCQ12 overall, prior aortic valve procedure, home oxygen, coronary artery disease presentation, prior 2-week HF, prior 2-week NYHA, prior cardiogenic shock, preoperative hemoglobin, and preoperative total albumin).

§ Derived from log-normal regression.

**Table 4:** Outcomes by follow-up survival status

Variable	Dead (n = 54)	Survival (n = 320)	*P* value∗
Postaortic valve perivalvular leak			.87
Mild	10 (37.0)	56 (34.4)	
Moderate	2 (7.4)	9 (5.5)	
None	15 (55.6)	98 (60.1)	
Missing	27	157	
5-m-walk test (sec)	10.0 ± 4.5	8.0 ± 4.4	**<.01**
Missing	27	102	
Event			**<.001**
None	16 (29.6)	209 (65.3)	
Cardiac	8 (14.8)	22 (6.9)	
Neurological	1 (1.9)	2 (0.6)	
Bleeding	1 (1.9)	3 (0.9)	
Vascular	0 (0.0)	4 (1.3)	
Renal	1 (1.9)	0 (0.0)	
Noncardiac readmission	26 (48.2)	80 (25.0)	
Single leaflet device attachment	1 (1.9)	0 (0.0)	

Values are presented as n (%) or mean ± SD. *Cardiac event*, Atrial fibrillation, conduction/native pacer, mitral valve reintervention, or myocardial infarction; *PCI*, percutaneous coronary intervention; *Neurological event*, ischemic stroke, transient ischemic attack, or undetermined stroke; *Bleeding event*, life threatening bleeding or major bleeding event. *Vascular event*, major vascular complication, unplanned vascular surgery, or intervention. *Renal event*, New requirement for dialysis.

∗ Derived from Wilcoxon rank-sum test for continuous variables and Fisher exact test for categorical variables.

**Table 5:** Identification of preoperative characteristics associated with follow-up mortality

Variable	Unadjusted		Adjusted†	
	Hazard ratio∗ (2.5%-97.5%)	*P* value	Hazard ratio (2.5%-97.5%)	*P* value
Age	1.024 (0.988-1.062)	.19		
Male	0.977 (0.572-1.669)	.93		
STS risk score	**1.122 (1.048-1.201)**	**<.001**	1.03 (0.93-1.13)	.61
KCCQ12 overall	0.993 (0.981-1.005)	.24		
Weight	0.989 (0.973-1.005)	.18		
Pacemaker	1.191 (0.532-2.665)	.67		
Prior PCI	**2.086 (1.168-3.726)**	**.01**	1.22 (0.56-2.66)	.62
Prior CABG	1.031 (0.545-1.949)	.93		
Prior aortic valve Procedure	1.138 (0.622-2.082)	.67		
Preoperative AV: Replace	0.521 (0.126-2.163)	.37		
Preoperative MV: Replace	1.550 (0.214-11.212)	.66		
Preoperative MV: Repair	1.917 (0.467-7.878)	.37		
Prior medical history				
Stroke	1.484 (0.738-2.984)	.27		
PAD	**1.833 (1.021-3.290)**	**.04**	1.74 (0.83-3.64)	.14
Smoker	1.448 (0.519-4.037)	.48		
HTN	1.285 (0.312-5.297)	.73		
DM	**2.968 (1.641-5.366)**	**<.001**	**2.53 (1.18-5.43)**	**.02**
Current dialysis	**3.324 (1.029-10.732)**	**<.045**	3.12 (0.64-15.26)	.16
Chronic lung disease	1.318 (0.772-2.251)	.31		
Home oxygen	**2.015 (1.105-3.676)**	**.02**	1.16 (0.50-2.70)	.73
Immune suppressive	**2.236 (1.044-4.786)**	**.04**	1.71 (0.65-4.46)	.28
CAD presentation‡		.55		
STEMI	NA	NA		
Angina	1.299 (0.442-3.822)	.63		
Symptoms unlikely to be ischemic	0.687 (0.371-1.271)	.23		
Unstable angina	1.331 (0.396-4.474)	.64		
Prior MI	**2.129 (1.200-3.778)**	**.01**	1.92 (0.91-4.06)	.09
Prior 2-wk HF	1.049 (0.469-2.343)	.91		
Prior 2-wk NYHA§		.79		
Class II	0.875 (0.254-3.009)	.83		
Class III	1.341 (0.582-3.086)	.49		
Class IV	1.131 (0.430-2.974)	.80		
Afib/flutter	**1.905 (1.078-3.367)**	**.03**	**2.04 (1.01-4.11)**	**<.0**47
AV morphology‖		.25		
Tricuspid	**0.229 (0.055-0.944)**	**.04**		
Uncertain	0.099 (0.009-1.119)	.06		
Unicuspid	NA	NA	NA	NA
Valve in valve	0.456 (0.110-1.895)	.28		
Preoperative hemoglobin	**0.819 (0.709-0.947)**	**<.01**	0.88 (0.71-1.10)	.25
Preoperative creatinine	1.256 (0.937-1.684)	.13		
Total albumin	**0.549 (0.334-0.904)**	**.02**	1.12 (0.54-2.32)	.76
LVEF	0.985 (0.966-1.004)	.12		
LVESD	0.816 (0.567-1.174)	.27		
LVEDD	0.832 (0.605-1.145)	.26		
5-m walk test¶		**<.01**		.05
>7 sec	2.213 (0.986-4.965)	.05	1.75 (0.71-4.32)	.23
Unable to walk	**4.651 (1.924-11.240)**	**<.001**	**3.52 (1.27-9.78)**	**.02**

*STS*, Society of Thoracic Surgeons; *KCCQ12*, Kansas City Cardiomyopathy Questionnaire 12; *PCI*, percutaneous coronary intervention; *CABG*, coronary artery bypass grafting; *AV*, aortic valve; *MV*, mitral valve; *PAD*, peripheral artery disease; *HTN*, hypertension; *DM*, diabetes mellitus; *CAD*, coronary artery disease; *STEMI*, ST-elevation myocardial infarction; *MI*, myocardial infarction; *HF*, heart failure; *NYHA*, New York Heart Association; *AFib*, atrial fibrillation; *LVEF*, left ventricular ejection fraction; *LVESD*, left ventricular end systolic dimension; *LVEDD*, left ventricular end diastolic dimension.

∗ Hazard ratio derived from Cox regression.

† Adjusted for the variables with an unadjusted *P* < .05.

‡ Versus no symptoms, no angina.

§ Versus I.

‖ Versus bicuspid.

¶ Versus ≤7 sec.

On subanalysis of patients who were able to perform the 5-m walk test, the hazard ratio (Group S vs Group N) was 2.22 (95% CI, 0.99-4.99; *P* = .053) without adjusting for potential confounders and 1.96 (95% CI, 0.85-4.51; *P* = .11) after adjusting the variables, which were significantly associated with survival (including diabetes, current dialysis, hemoglobin level before the procedure, and creatinine level before the procedure). When the 5-m walk test result (time) was treated as a continuous variable, the hazard ratio for every second increase was 1.07 (95% CI, 1.01-1.14; *P* = .02) without adjusting for potential confounders and 1.09 (95% CI, 1.02-1.16; *P* = .02) after adjusting the variables, which were significantly associated with survival (including diabetes, current dialysis, hemoglobin level before the procedure, and creatinine level before the procedure). This indicates that the relationship was not solely driven by those who could not walk (Tables E2 and E3).

**Figure E1:**
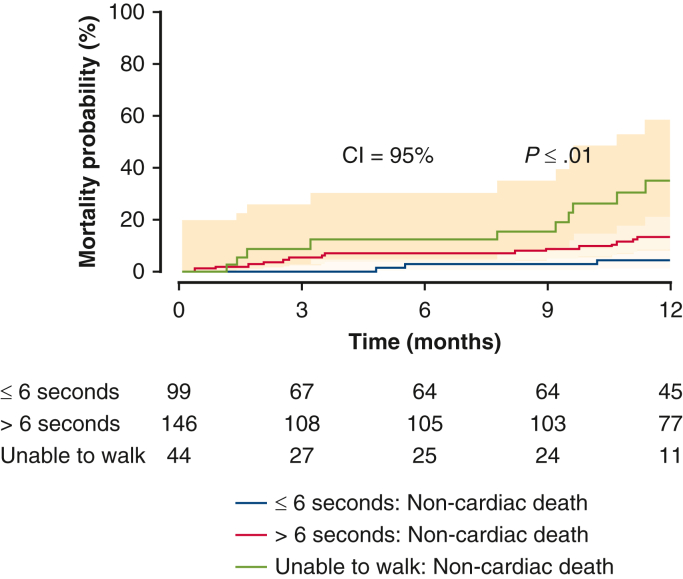
Noncardiac mortality after transcatheter aortic valve replacement by the 5-m walk test (6 seconds). The patients who were unable to walk before transcatheter aortic valve replacement had significantly worse noncardiac mortality than patients who walked 5 m in <6 seconds or who walked 5 m in >6 seconds (*P* < .01). Ninety five percent confidence limit was used.

**Figure E2:**
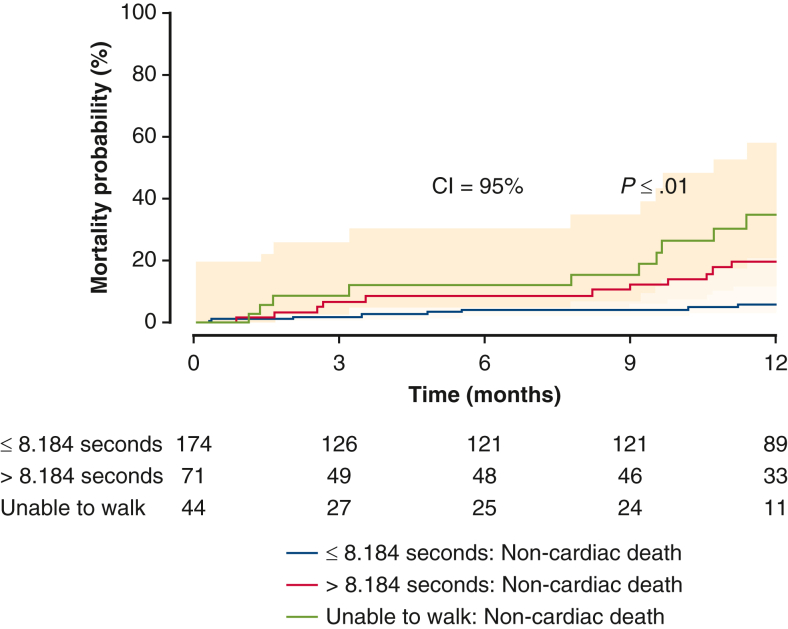
Noncardiac mortality after transcatheter aortic valve replacement by the 5-m walk test (8.184 seconds). The patients who were unable to walk before transcatheter aortic valve replacement had significantly worse noncardiac mortality than patients who walked 5 m in <8.184 seconds or who walked 5 m in >8.184 seconds (*P* < .01). Ninety five percent confidence limit was used.

**Figure E3:**
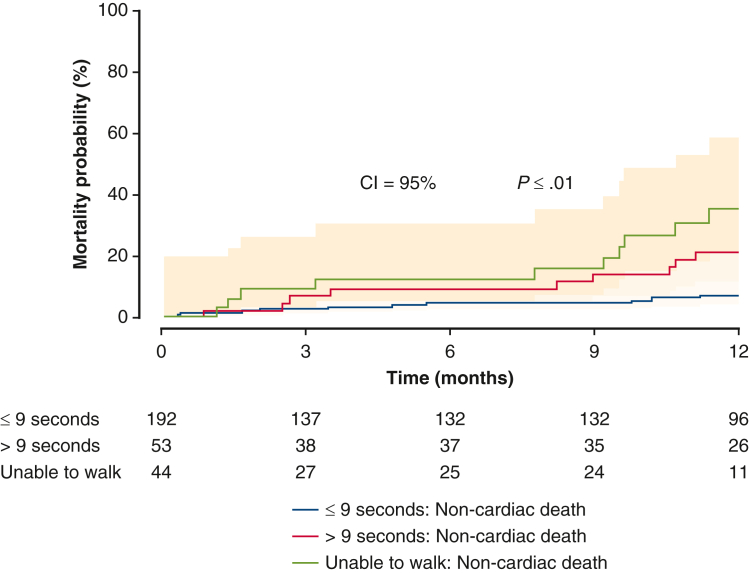
Noncardiac mortality after transcatheter aortic valve replacement by the 5-m walk test (9 seconds). The patients who were unable to walk before transcatheter aortic valve replacement had significantly worse noncardiac mortality than patients who walked 5 m in <9 seconds or who walked 5 m in >9 seconds (*P* < .01). Ninety five percent confidence limit was used.

Regarding the relationship between 3 additional 5-m-walk test cutoffs and 1-year noncardiac mortality, we found that same results (Figure E1, Figure E2, Figure E3).

There was no evidence indicating that the proportional hazard assumption was violated (global *P* = .18 for all the variables in the adjusted model; *P* = .75 for the 5-m walk test group) based on examining Schoenfeld score residuals.

## Discussion

**Figure 3:**
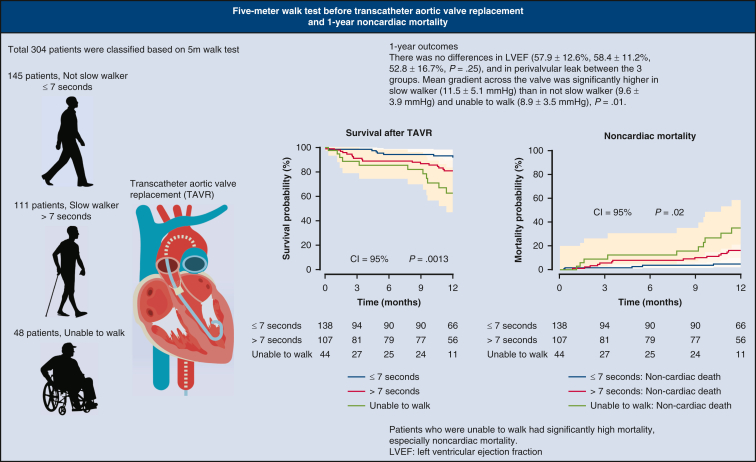
Key findings of the study. A 5-m walk test score before transcatheter aortic valve replacement was an independent risk factor for 1-year noncardiac mortality. *LVEF*, Left ventricular ejection fraction.

This study demonstrated that the pre-TAVR 5-m walk test was an independent risk factor for 1-year noncardiac mortality despite satisfactory results at 1-year follow-up, including mean gradient across the valve, perivalvular leak, and LVEF. The patients in Group N had the highest survival rate compared with Group S and Group I (Figure 3).

The 5-m walk test has been utilized to assess periprocedural risk on both adult cardiac surgery and TAVR.^15^^,^^16^ It is among the components of frailty assessments, including a hand grip measurement, weight loss, exhaustion, depression, low physical activity, physical activity index, cognitive impairment, a side-by-side, semitandem, and tandem stand balance tests, the repeated chair stand test, hemoglobin level, and serum albumin level.^13^^,^^17^, ^18^, ^19^ The 5-m walk test is an important indicator of sarcopenia or the loss of muscle tissue due to aging.^20^ Poor preprocedural performance has been reported as a risk factor for poor outcomes.^16^ Although it covers only 1 aspect of frailty, it is simple to perform in the busy clinical setting and a relatively reliable way to assess frailty.

The recent study suggests that those with an increasing number of positive markers of frailty (eg, the 5-m walk test, anemia, and low albumin level) are at an increased risk of mortality after TAVR.^13^ It indicates that those patients who developed a frail status from severe AS missed the optimal timing for intervention and, consequently, experienced some organ damage. Another potential explanation is that such patients already lost their physiologic reserve from multiple comorbidities. Our study demonstrates that the rate of mortality is significantly higher in Group S and Group I compared with the Group N. As opposed to the previous study, preprocedural serum albumin level or preprocedural anemia were not risk factors for poor survival at 1 year (Table 5). It is possible that our sample size may not be large enough to detect these as risk factors for poor survival at 1 year, which may suggest that the 5-m walk test may be a more sensitive indicator or early sign of frailty than either serum albumin level or preprocedural anemia. Procedure-related success, aortic valve function, and LVEF were not risk factors for poor outcomes. Previous studies have demonstrated that TAVR helps prolong the life of a patient with severe AS.^1^^,^^7^^,^^8^ However, a comparison between the outcomes of survivors and nonsurvivors, shows that the baseline physiologic reserve had a more significant influence on 1-year survival than overall aortic valve function.

Furthermore, we found that patients in the slow-walker and unable-to-walk groups had higher STS-PROM scores and lower KCCQ12. This is expected given that these patients are frailer at baseline, and these scores are independently associated with worse outcomes in cardiac patients. To discern the effect of preprocedural characteristics and the 5-m walk test on 1-year mortality, we performed a subanalysis on the patients who were able to walk. It demonstrated that the 5-m walk test score was significantly associated with 1-year survival after adjusting the variables. Group S and Group I had lower preoperative hemoglobin levels. This is consistent with the systematic analysis of a former study^21^ that found that preoperative anemia is independently associated with higher odds of long-term mortality in patients undergoing TAVR (odds ratio, 1.77; 95% CI, 1.34-2.35). They did not, however, find an association between preoperative anemia and short-term (30 days) mortality (odds ratio, 1.34; 95% CI, 0.77-2.35). In terms of preoperative albumin levels, patients in the slow-walker and unable-to-walk groups had lower preoperative albumin levels. It is well established in the literature that frail patients are more likely to have low albumin levels and tend to have worse outcomes.^22^^,^^23^ Furthermore, albumin level is a tool that has been used extensively as an adjunct in frailty assessment scores. In terms of preexisting comorbidities, Group S and Group I had higher rates of prior aortic interventions and diabetes compared with Group N. The higher incidence of prior aortic interventions and diabetes in these patients is consistent with the previous report.^24^

**Figure E4:**
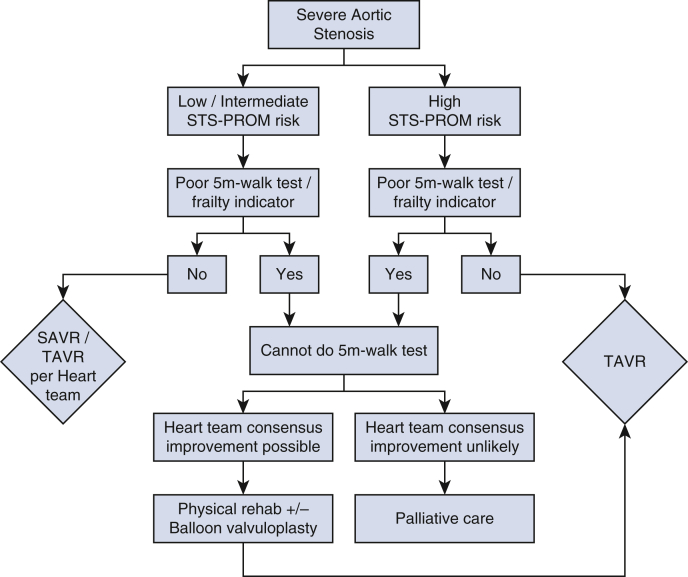
Management of severe aortic valve stenosis for transcatheter aortic valve replacement. The patients who are not able to walk before transcatheter aortic valve replacement undergo medical and physical optimization. *STS-PROM*, Society of Thoracic Surgeons predicted risk of mortality; *SAVR*, surgical aortic valve replacement; *TAVR*, transcatheter aortic valve replacement.

We believe our study demonstrates that patients should proceed with TAVR at an earlier stage of physical status decline before developing a nonambulatory state. Given that physical impairment happens quickly after developing typical severe AS symptoms,^25^ such patients must be monitored more closely to proceed with TAVR without delay. In addition, given there are some reports on early AVR being associated with improved survival in asymptomatic AS,^26^^,^^27^ the 5-m walk test may help determine the timing of intervention. Furthermore, such information helps clinicians provide realistic expectations to nonambulatory patients and their families. For a patient who takes more than 7 seconds to walk 5 m or who is in a nonambulatory state, conducting preprocedural rehabilitation may reverse frailty and obtain better 1-year survival. In our institution, we try to optimize patients who are not able to walk 5 m before the TAVR (Figure E4). In addition, this finding may be applied to patient selection for surgical AVR. Even if patients are young and have a low STS-PROM score, slower walkers may be better with TAVR.

Our study has some limitations that are mostly attributable to the retrospective nature of the analysis and the effect of unmeasurable confounding factors that cannot be captured, such as previous major medical issues, injuries, and socioeconomic status. Being a single-center study with a small sample size limits the generalizability of the results. However, the strength of our study lies in using a single frailty marker to predict outcomes and its long-term 1-year follow-up.

## Conclusions

The pre-TAVR 5-m walk test score was associated with 1-year mortality, especially noncardiac mortality. This score may help identify patients with a higher 1-year mortality post-TAVR and aid in preoperative discussion and recovery expectations (Video 1).

### Conflict of Interest Statement

The authors reported no conflicts of interest.

The *Journal* policy requires editors and reviewers to disclose conflicts of interest and to decline handling or reviewing manuscripts for which they may have a conflict of interest. The editors and reviewers of this article have no conflicts of interest.

## Figures and Tables

**Table E1:** Relationship between noncardiac death and the 5-m walk test result

5-m walk test result	Frequency (%) of noncardiac death	Unadjusted		Adjusted†	
		Hazard ratio (95% CI)∗	*P* value	Hazard ratio (95% CI)	*P* value
≤7 sec (n = 138)	7 (5.1)	Ref		Ref	
>7 sec (n = 107)	**14 (13.1)**	**2.65 (1.04-6.71)**	**.04**	2.30 (0.88-6.04)	.09
Unable to walk (n = 44)	**10 (22.7)**	**6.21 (2.30-16.78)**	**<.001**	**4.06 (1.19-13.87)**	**.03**

*CI*, Confidence limit; *Ref*, reference category.

∗ Derived from subdistribution hazard regression accounting for competing risks (such as cardiac death).

† Adjusted for variables associated with follow-up survival (Table 5).

**Table E2:** Identification of preoperative characteristics associated with follow-up survival (1 year)

Variable	Hazard ratio (2.5%-97.5%)	*P* value
Age	1.021 (0.974-1.070)	.39
Male	0.981 (0.457-2.103)	.96
STS risk score	1.109 (1.005-1.223)	.039
KCCQ12, overall	0.992 (0.977-1.007)	.32
Weight	0.984 (0.963-1.005)	.14
Pacemaker, yes	1.731 (0.649-4.618)	.27
Prior PCI, yes	1.343 (0.629-2.867)	.45
Prior CABG, yes	0.496 (0.171-1.434)	.2
Prior aortic valve, yes	1.033 (0.447-2.387)	.94
Prev Proc AV replace, yes	0.385 (0.051-2.883)	.35
Prev Proc MV replace, yes	2.292 (0.310-16.918)	.42
Prev Proc MV repair, yes	1.760 (0.238-13.001)	.58
Prior stroke, yes	2.111 (0.921-4.841)	.078
Prior PAD, yes	1.450 (0.651-3.229)	.36
Smoker, yes	0.000 (0.000-Inf)	>.99
HTN, yes	0.841 (0.199-3.558)	.81
DM, yes	2.478 (1.159-5.297)	.019
Current dialysis, yes	8.679 (2.038-36.951)	.003
Chronic lung disease, yes	1.004 (0.457-2.207)	>.99
Home oxygen, yes	1.764 (0.743-4.188)	.2
Immunosuppression, yes	2.689 (1.013-7.134)	.047
CAD presentation: Non-STEMI	0.000 (0.000-Inf)	>.99
CAD presentation: Stable angina	1.199 (0.343-4.192)	.78
CAD presentation: Symptom unlikely to be ischemic	0.407 (0.172-0.964)	.041
CAD presentation: Unstable angina	0.784 (0.103-5.947)	.81
Prior MI, yes	1.935 (0.888-4.215)	.097
Prior 2-wk HF, yes	2.195 (0.518-9.297)	.29
Prior 2-wk NYHA class II	1.135 (0.158-8.131)	.9
Prior 2-wk NYHA class III	2.846 (0.658-12.313)	.16
Prior 2-wk NYHA class IV	2.056 (0.399-10.608)	.39
AFib/flutter, yes	2.023 (0.943-4.340)	.07
AV morphology: Tricuspid	0.155 (0.036-0.658)	.012
AV morphology: Uncertain	0.097 (0.008-1.124)	.062
AV morphology: Unicuspid	0.000 (0.000-Inf)	>.99
Valve in valve, yes	0.340 (0.046-2.535)	.29
Preprocedure hemoglobin	0.809 (0.659-0.994)	.043
Preprocedure creatinine	1.717 (1.127-2.614)	.012
Total albumin	0.595 (0.291-1.215)	.15
LVEF	0.981 (0.957-1.006)	.13
LVESD	0.808 (0.506-1.290)	.37
LVEDD	0.861 (0.572-1.296)	.47
5-m-walk >7 sec	2.222 (0.990-4.987)	.053
5-m walk, continuous	**1.073 (1.010-1.139)**	**.022**

*STS*, Society of Thoracic Surgeons; *KCC Q12*, Kansas city cardiomyopathy questionnaire 12; *PCI*, percutaneous coronary intervention; *CABG*, coronary artery bypass grafting; *AV*, aortic valve; *MV*, mitral valve; *PAD*, peripheral artery disease; *HTN*, hypertension; *DM*, diabetes mellitus; *CAD*, coronary artery disease; *STEMI*, ST-elevation myocardial infarction; *MI*, myocardial infarction; *HF*, heart failure; *NYHA*, New York Heart Association; *AFib*, atrial fibrillation; Aflutter, atrial flutter; *LVEF*, left ventricular ejection fraction; *LVESD*, left ventricular end systolic dimension; *LVEDD*, left ventricular end diastolic dimension.

**Table E3:** Adjusted analysis treating the 5-m walk test result as a continuous variable

Variable	Hazard ratio (2.5%-97.5%)	*P* value
Diabetes yes	2.731 (1.247-5.980)	.012
Current dialysis, yes	5.858 (0.543-63.179)	.15
Preprocedure hemoglobin	0.874 (0.701-1.091)	.23
Preprocedure creatinine	1.168 (0.620-2.200)	.63
5-m walk, continuous	**1.087 (1.015-1.163)**	**.017**

## Supplementary Data

Video 1 This video explains brief overview of the study protocol and major findings of the study. Video available at: https://www.jtcvs.org/article/S2666-2736(22)00313-8/fulltext.
