# Supplementary figures and images for: Five-meter walk test before transcatheter aortic valve replacement and 1-year noncardiac mortality
Source: JTCVS Open. 2022 Aug 18;12:103–17. doi: 10.1016/j.xjon.2022.08.003 (PMC9801278; doi:10.1016/j.xjon.2022.08.003)

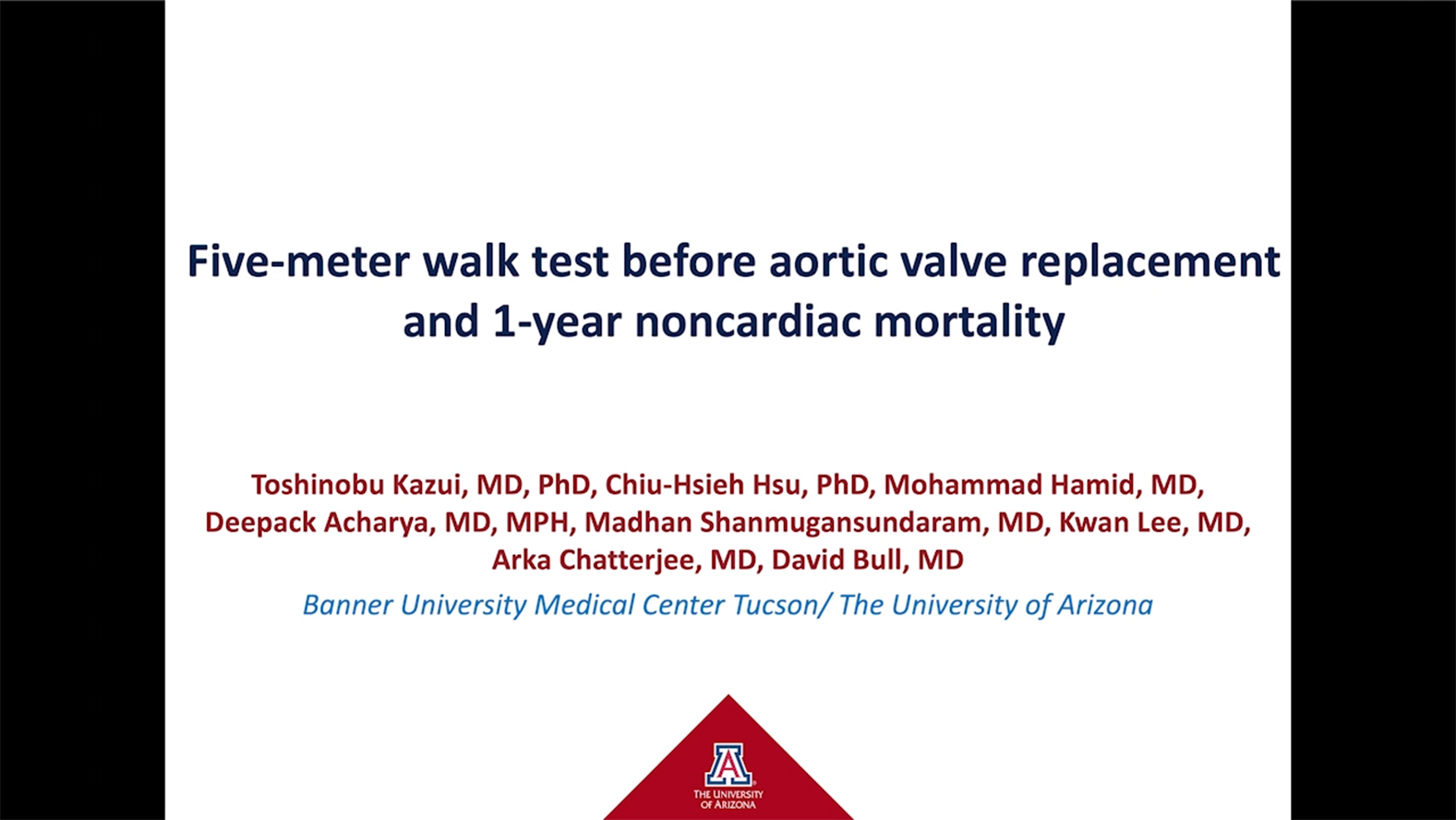

Supplement: Video 1 — This video explains brief overview of the study protocol and major findings of the study. Video available at: https://www.jtcvs.org/article/S2666-2736(22)00313-8/fulltext. [file fx3.jpg]
